# miR-100 inhibits cell proliferation in mantle cell lymphoma by targeting mTOR

**DOI:** 10.1186/s40164-020-00182-2

**Published:** 2020-09-26

**Authors:** Luhui Lin, Yiqun Huang, Wei Zhuang, Ping Lin, Xudong Ma

**Affiliations:** 1 Department of Hematology, Zhangzhou Affiliated Hospital of Fujian Medical University, Zhangzhou, Fujian China; 2 Graduate School, Fujian Medical University, Fuzhou, Fujian China

**Keywords:** miR-100, mTOR, Mantle cell lymphoma, Double luciferase assay

## Abstract

**Background:**

miR-100 is reported to be associated with cell proliferation and apoptosis. However, the function of miR-100 in mantle cell lymphoma (MCL) is unknown. The purpose of this study is to analyze the abnormal expression of miR-100 and mTOR in MCL together with their potential biological function and pathogenesis.

**Method:**

Eighteen MCL tissue samples and 3 cell lines (Jeko-1, Mino, Granta-519) were investigated in this research study, while eighteen samples of proliferative lymphadenitis from patients and peripheral lymphocyte cells from healthy volunteers served as controls. The expression and alteration of miR-100 and mTOR mRNA were detected by RT-PCR. The expression and alteration of mTOR protein were explored by Western blot. LV-miR-100-up and LV-mTOR-RNAi were constructed and transfected by lentivirus transfection. Cell proliferation, cell apoptosis and the cell cycle were detected using CCK-8 and flow cytometry. Bioinformatics prediction software was used to predict the miR-100 target gene of mTOR. A double luciferase experiment was used to verify miR-100 targeting at the mTOR-3′-UTR. The interaction between miR-100 and mTOR was further studied using recovery experiments. GraphPad Prism 7 software (version 7.2) was used for statistical analysis, and a *P* value < 0.05 was considered statistically significant.

**Results:**

We found that the expression of miR-100 mRNA in MCL tissues and cell lines was lower, while that of the mTOR protein was higher. There was a negative correlation between miR-100 and mTOR in both MCL tissues and cell lines. Promoting miR-100 and inhibiting mTOR could inhibit cell proliferation, induce cell apoptosis and block the cell cycle in the G1 phase. A double luciferase reporter assay showed that mTOR was one of the target genes of miR-100. The recovery experiment demonstrated that PV-mTOR-up partially set off the effect of LV-miR-100-up on decreasing mTOR expression, inhibiting proliferation, inducing apoptosis and blocking the cell cycle in G1 phase in both Jeko-1 and Mino cells.

**Conclusions:**

Abnormal expression of miR-100 and mTOR was found in MCL, which included downregulation of miR-100 and upregulation of mTOR. The expression of mTOR is negatively correlated with miR-100. It may play an important role in MCL pathogenesis. miR-100 up-regulation can inhibit cell proliferation, promote cell apoptosis, and inhibit cell cycle in G1 phase by targeting the mTOR gene. miR-100 may potentially be an anti-mantle cell lymphoma gene.

## Introduction

Mantle cell lymphoma (MCL) is an invasive non-Hodgkin lymphoma (NHL) derived from B cells, accounting for approximately 7% of the NHL incidence rate [1]. The genetic feature of MCL is the chromosomal translocation t (11;14) (q13; q32), resulting in overexpression of cyclin D1. The efficacy of radiotherapy and chemotherapy is limited. The overall survival of patients with MCL is 4 ~ 5 years [2].

miRNAs are a type of small noncoding RNA with a length of 22 ~ 24 nucleotides that adjust to match the target gene mRNA 3′-end untranslated region (3′-UTR). miRNAs are currently a research hotspot in the process of malignant tumor development, diagnosis, prognosis, drug resistance and treatment. miR-100 is located at 11q24.1 in the human genome, with the gene sequence AACCCGUAGAUCCGAACUUGUG. miR-100 can be a tumor suppressor gene or oncogene, and its expression and function vary in different tumors, and sometimes have opposing roles. It plays a dual role in cancer, according to the changes caused by target genes regulated in related tumors and could even affect tumor biological behavior.

mTOR is a major downstream factor in the PI3K/AKT signaling pathway, which is highly expressed in many different kinds of tumors [3]. Xie et al. [4] found an important role of the mTOR pathway in the tumorigenesis of MCL. Hess et al. [5] determined that temsirolimus, an inhibitor of mTOR, was beneficial in all MIPI risk categories in patients with MCL. With the discovery of miR-100, an increasing number of related studies have been conducted, some of which have identified target genes and the corresponding signaling pathways of miR-100. Biological information predicted that the mTOR-encoding gene might be one of the target genes of miR-100. In acute lymphoblastic leukemia, Li et al. [6] found that miR-100 could inhibit cell proliferation and promote cell apoptosis by regulating the target gene FKBP51, affecting glucocorticoid receptor activity, or inhibiting the expression of IGFIR and mTOR. Sun et al. [7] showed that miR-99a/100 promoted apoptosis by targeting mTOR in esophageal squamous cell carcinoma cells. Derynck et al. [8] demonstrated that miR-100 might participate in the epithelial-mesenchymal transformation of hepatocellular carcinoma cells by regulating the expression of mTOR and affecting hepatocellular carcinoma cell invasion and metastasis. Xu et al. [9] also suggested that miR-100 is involved in tumorigenesis and development by regulating the expression of the target gene mTOR. In summary, the abnormal expression of the mTOR pathway promoted the development of MCL, and mTOR was regulated by miR-100 in a variety of different tumors, resulting in the alteration of biological functions, such as cell proliferation, apoptosis, invasion and metastasis.

However, there is limited understanding of the miR-100 regulatory mechanisms in MCL and the corresponding regulation of the mTOR target gene. In this study, we investigated the expression patterns of miR-100 and mTOR in MCL. Furthermore, the roles of miR-100 and mTOR in proliferation, apoptosis and cell cycle progression were studied in MCL cell lines. Direct targets of miR-100 were identified to elucidate the possible regulatory mechanism of miR-100 in MCL.

## Materials and methods

### Human tissues and cell lines

Eighteen human MCL specimens were obtained from resection tissues at Zhangzhou Affiliated Hospital of Fujian Medical University between Sep 2016 and Apr 2018. At the same time, 18 patients with hyperplastic lymphadenitis were used as the control. All MCL diagnoses were confirmed by an experienced pathologist on the basis of morphology, immunophenotype findings and molecular genetics. Patients did not undergo any radiotherapy or chemotherapy before resection. The tissue specimens were frozen immediately in liquid nitrogen and stored at −80 °C. This study was conducted in accordance with the Declaration of Helsinki and supported by the Research Ethics Committee of Zhangzhou Affiliated Hospital of Fujian Medical University. Informed consent was obtained from all the participants before any clinical procedure was initiated.

Normal lymphoid B cells were collected from the blood of healthy volunteers. HEK293T cells and human MCL cell lines (Jeko-1, Mino, Granta-519) were cultured in RPMI‑1640 medium containing 15% FBS and 1% penicillin‑streptomycin. The experiments were conducted in the logarithmic phase and harvested at different time points.

### Lentivirus production and transduction

Lentiviral transduction was utilized to overexpress miR-100 in MCL cell lines (Jeko-1, Mino). The lentiviruses contained human miR-100-up-GFP (LV-miR-up), the control (LV-NC-up), LV-mTOR-RNAi-GFP (LV-mTOR-RNAi) and the control (LV-NC-RNAi). The plasmids containing the 3′-UTR of the mTOR luciferase plasmid, miR-100 plasmid, Renilla plasmid, and mTOR-up-GFP plasmid were purchased from Genechem Company (Genechem Co, Ltd, Shanghai, China). The MOI of Jeko-1 was 40, and the MOI of Mino was 60. The lentiviruses were added to 2 × 10^5^/mL cells in 6-well plates with 1 ml of RPMI‑1640 containing no FBS and 40 μl/mL HitransG (Genechem Co, Ltd, Shanghai, China). Fresh culture medium contained 15% FBS after 16 h. Cells were harvested after 72 h of transduction, and qRT‑PCR was performed to confirm the efficiency of transduction.

### Bioinformatics prediction and dual‑luciferase reporter assay

The present study used algorithms for target gene prediction. miRanda (https://www.microrna.org/microrna/home.do), TargetScan (https://www.targetscan.org/) and PicTar (https://pictar.mdc-berlin.de/) predicted the potential target genes of miR‑100. Next, a dual‑luciferase reporter assay was performed to validate the predicted targets. HEK293T (1 × 10^5^/mL) cells were inoculated in 24‑well plates and cultured in a 37 °C and 5% CO_2_ incubator. Wild‑type (WT) and mutant (MUT) mTOR were cloned into a renilla plasmid to construct mTOR‑WT and mTOR‑MUT vectors, respectively. Subsequently, 0.1 μg of the 3′-UTR of mTOR luciferase plasmid, 0.4 μg of the miR-100 plasmid, and 0.02 μg of the renilla plasmid were co‑transfected into HEK293T cells using X-tremegene HP transfection reagent. Luciferase activity was measured using the Dual‑Luciferase Reporter System (Promega Corporation, Madison, WI, USA) at 48 h after transfection.

### RT-PCR

Total RNA was extracted from tissues and cells using TRIzol reagent (Invitrogen Life Technologies, Carlsbad, CA, USA) according to the manufacturer's instructions. The RNA was reverse transcribed into cDNA using the Prime Script RT Reagent Kit (Promega Corporation, Madison, WI, USA) with cDNA as the template. RT-PCR was performed using the SYBR green PCR assay (Takara, People's Republic of China). The primers for miR-100, U6, mTOR and GAPDH were purchased from Genechem Company (Genechem Co, Ltd, Shanghai, China). The PCR conditions consisted of 95 °C pre‑denaturation for 10 min, followed by 40 cycles at 95 °C for 5 s, 60 °C for 20 s, and extension at 72 °C for 15 s. qRT-PCR was performed on the Bio‑Rad CFX96/CFX Connect™ system (Bio‑Rad Laboratories, Inc, Hercules, CA, USA) to test the relative expression.

### Western blot assay

The cell precipitate was washed twice with precooled PBS and lysed by adding 100 μL of lysate and 1 μL of enzyme inhibitor (Sigma-Aldrich, St. Louis, MO, USA) for 30 min on ice. After centrifugation at 10,000 × *g* at 10 °C for 10 min, the protein was quantified by a BCA protein assay kit (Sigma-Aldrich, St. Louis, MO, USA). A total of 50 µg of protein was separated by 12% SDS-PAGE electrophoresis and transferred to a membrane. After blocking at room temperature for 1 h, the membrane was incubated with rabbit monoclonal antibodies against mTOR (1:2000, Abcam USA, Cambridge, MA, USA) and mouse monoclonal antibodies against GAPDH (1:2000, Abcam USA, Cambridge, MA, USA) at 4 °C overnight. The membrane was washed with TBS and incubated with HRP-conjugated goat anti-mouse or anti-rabbit secondary antibody at 1:2000 (Abcam USA, Cambridge, MA, USA). Finally, the membrane was developed and analyzed using Image analysis software.

### Cell proliferation assay

Cell viability was detected using the Cell Counting Kit-8 (CCK-8, Sigma-Aldrich, St, Louis, USA) assay according to the manufacturer’s protocol. In brief, cells were plated in a 96-well plate at 2 × 10^5^/mL per well and infected with miR-100-up or mTOR-RNAi and the corresponding NC lentivirus. Cell proliferation was determined by the CCK-8 assay at the indicated time points. Ten microliters of CCK-8 reagent were added to each well. The absorption (A value) was measured at 450 nm wavelength on the enzyme label to calculate the cell survival rate, using the following equation: (%) = [A (medication) − A (blank)]/[A (control) − A (blank)] × 100. The experiment was performed in triplicate.

### Cell apoptosis assay

Cells were plated in 6-well plates at 2 × 10^5^/mL per well and infected with miR-100-up or mTOR-RNAi and the corresponding NC lentivirus for 6 days. The cells (2 × 10^6^/mL) were collected in a 5 mL centrifuge tube and centrifuged at 1300 rpm for 5 min. The supernatant was discarded. The cell pellet was washed with D-Hanks precooled at 4 °C, washed once with 1 × binding buffer, and centrifuged at 1300 rpm for 3 min to collect the cells. The cell pellet was resuspended in 200 µL 1 × binding buffer. Cells were incubated in 5 µL of Annexin V‑FITC and 5 µL of PI in the dark for 10 min. Next, the cells were resuspended in 400 µL of binding buffer. Cell apoptosis was measured using an apoptosis kit (BD Biosciences, San Jose, CA, USA) according to the manufacturer's instructions.

### Cell cycle assay

The cell cycle was measured using PI (Sigma-Aldrich, St, Louis, USA). Cells were plated in 6-well plates at 2 × 10^5^/mL per well and infected with miR-100-up or mTOR-RNAi and the corresponding NC lentivirus for 6 days. The cells were collected in a 5 mL centrifuge tube and centrifuged at 1300 rpm for 5 min. The supernatant was discarded. The cell pellet was washed with D-Hanks precooled at 4 °C and centrifuged at 1300 rpm for 5 min. The 75% ethanol was precooled and incubated with the cells for at least 1 h. The samples were centrifuged at 1300 rpm for 5 min to remove the fixative. Then, the cell pellet was washed once with D-Hanks. Preparation of cell staining solution included the following calculations: 40 × PI mother liquor (2 mg/mL): 100 × RNase mother liquor (10 mg/mL): 1 × D-Hanks = 25:10:1000. Analysis was performed using flow cytometry analysis software.

### Statistical analysis

Statistical analysis was performed using GraphPad Prism version 7.2. Data are presented as the mean ± SEM. Data were analyzed using an unpaired *t* test for comparisons of two cohorts. One-way ANOVA was used to analyze the remaining data. Linear regression was used for correlation analysis. *p* < 0.05 was considered statistically significant.

## Results

### The poor expression of miR-100 in MCL tissues and cell lines

**Fig. 1:**
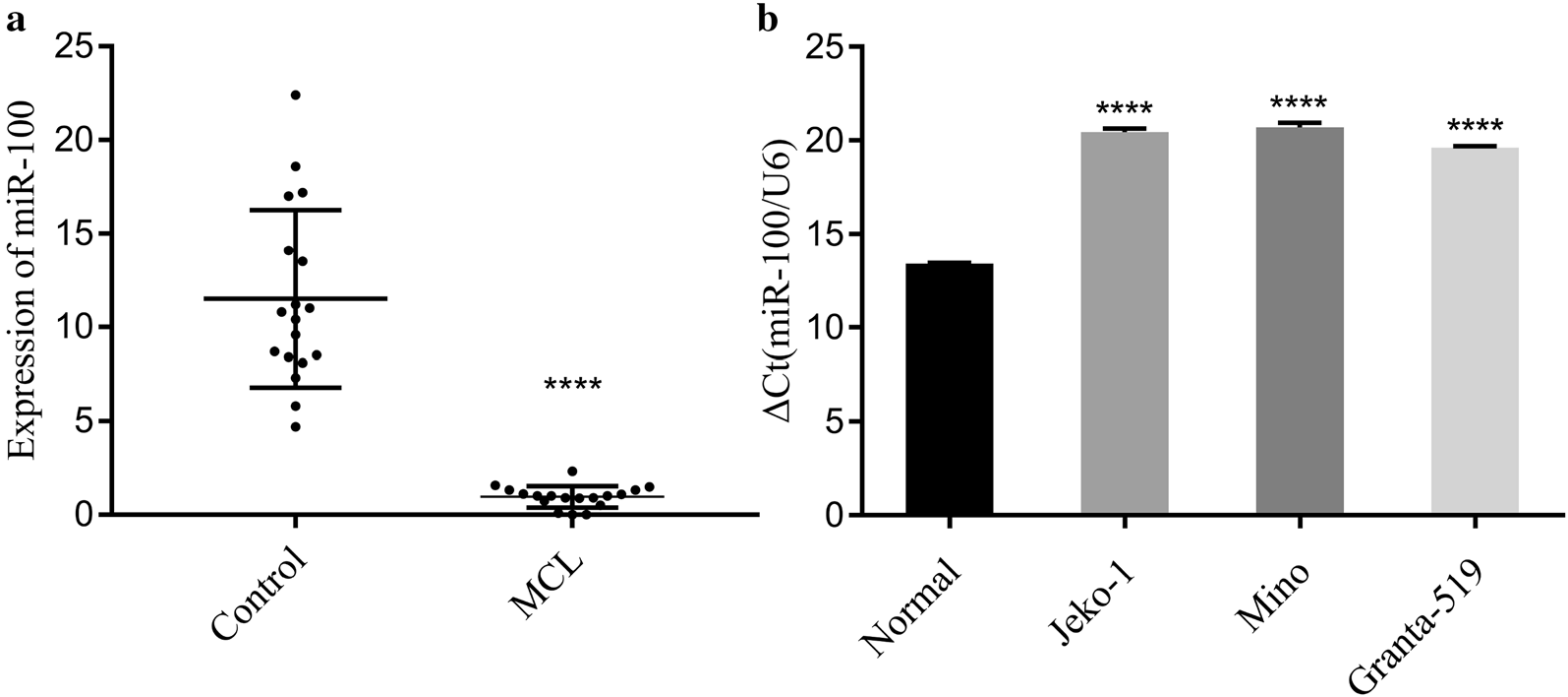
The abnormal expression of miR‑100 in MCL tissues and cell lines. **a** RT‑PCR showed that the expression of miR‑100 was reduced in MCL compared to that in hyperplastic lymphadenitis (*p* < 0.0001). **b** The RT‑PCR results showed that the expression of miR‑100 was reduced in MCL cells compared to peripheral lymphocyte cells (*p* < 0.0001)

To evaluate the state of miR-100 in MCL, the expression of miR-100 in MCL tissues and cell lines was examined by using RT-PCR. miR-100 mRNA was reduced in MCL both in tissues and cell lines compared to hyperplastic lymphadenitis and peripheral lymphocyte cells (*p* < 0.0001, Fig. 1a, b). This result indicated that miR-100 downregulation might be associated with the formation of MCL. The relationship between clinical features and the level of miR-100 expression was analyzed by the χ^2^ test, which demonstrated that the lower expression of miR-100 was related to a higher LDH level (≥ 300 U/L) (*p* = 0.0085) and IPI score > 3 points (*p* = 0.0156). There was no relationship between patient age, gender, B symptoms, clinical stage, or bone marrow involvement (*p* > 0.05; Table 1).

**Table 1:** Relationship between expression of miR‑100 and clinical characteristics of MCL cases

Clinical features	n	High miR-100	Low miR-100	χ2	p value
Age (years)				0.9	0.3428
≤ 58	8	5	3		
>58	10	4	6		
Sex				0.2769	0.5987
Male	13	6	7		
Female	5	3	2		
B symptoms				2.25	0.1336
Present	2	0	2		
Absent	16	9	7		
LDH (U/L)				6.923	0.0085
<300	13	9	4		
≥ 300	5	0	5		
Clinical stage				3.6	0.0578
I–II	3	3	0		
III–IV	15	6	9		
IPI score				5.844	0.0156
<3	11	8	3		
≥ 3	7	1	6		
Involve bone marrow				0.2338	0.6287
Present	11	5	6		
Absent	7	4	3		

### High expression of mTOR in MCL tissues and cell lines

**Fig. 2:**
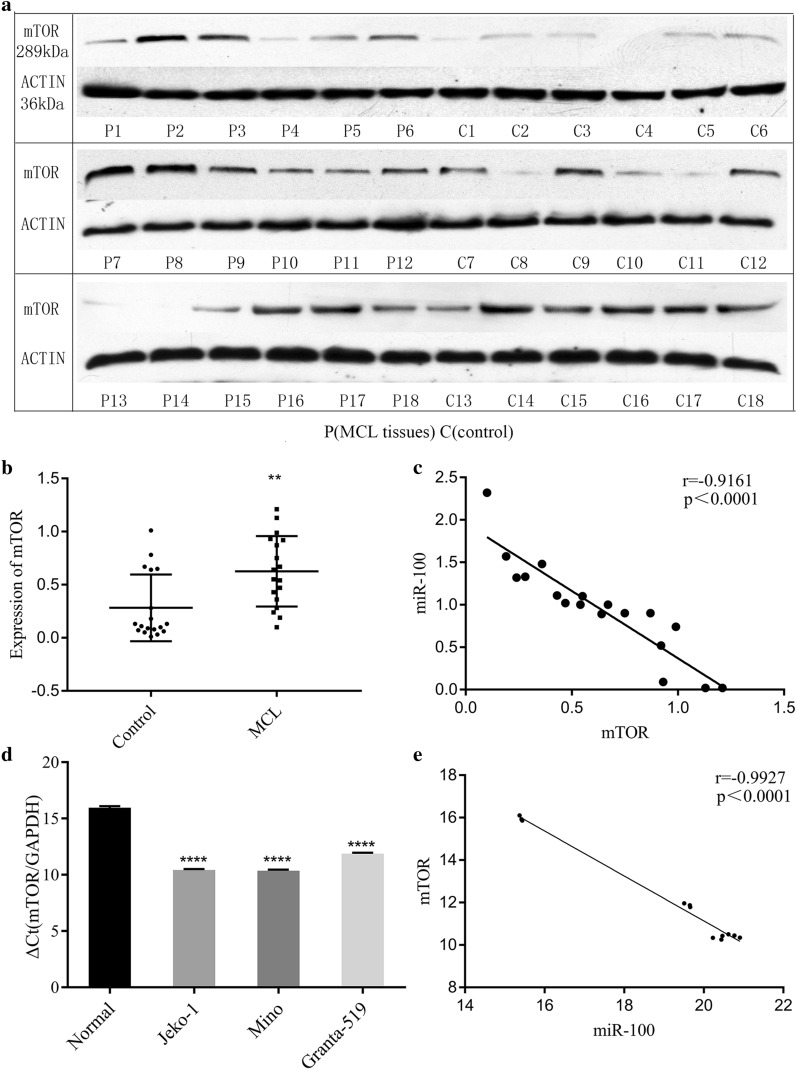
Evaluate the state of mTOR in MCL tissues and cell lines. **a** mTOR protein expression in MCL tissues and hyperplastic lymphadenitis. **b** Western blot analysis of mTOR protein upregulation in MCL compared to that in hyperplastic lymphadenitis (*p* = 0.003). **c** The expression of mTOR protein was negatively correlated with that of miR-100 mRNA in MCL tissues (r = −0.9196, *p* < 0.0001). **d** RT-PCR analysis showed that mTOR mRNA was higher in MCL cells than in peripheral lymphocyte cells (*p* < 0.0001). **e** The expression of mTOR mRNA was negatively correlated with that of miR-100 in MCL cells (r = −0.9927, *p* < 0.0001)

Western blotting detected mTOR protein expression in MCL tissues, and mTOR mRNA in cell lines was checked by RT-PCR. mTOR was overexpressed in the MCL tissues and cell lines compared to the control group (*p* = 0.003, *p* < 0.0001, Fig. 2a, b, d). The expression of mTOR protein was negatively correlated with miR-100 mRNA in MCL tissues (r = -0.9196, *p* < 0.0001, Fig. 2c). The expression of mTOR mRNA was negatively correlated with the expression of miR-100 mRNA in MCL cells (r = −0.9927, *p* < 0.0001, Fig. 2e).

### miR-100 inhibited mTOR mRNA and protein

**Fig. 3:**
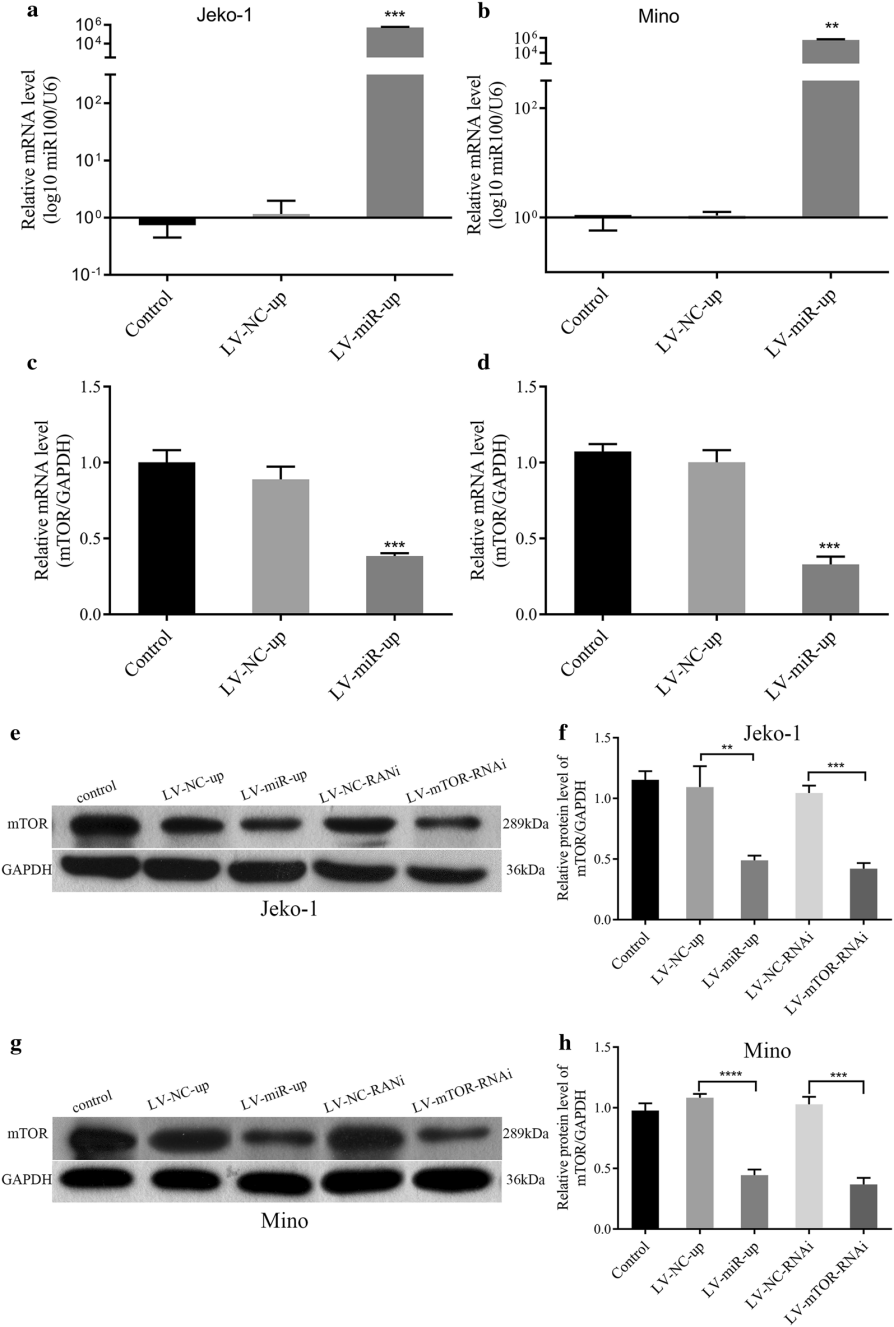
LV-miR-100 upregulated miR-100 and down regulated mTOR mRNA and protein levels. **a** RT-PCR showed that the miR-100 mRNA level was increased in the LV-miR-100-up group compared to the NC group in Jeko-1 cells (*p *= 0.0003). **b** RT-PCR showed that the miR-100 mRNA level was increased in the LV-miR-100-up group compared to the NC group in Mino cells (*p *= 0.0013). **c** RT-PCR showed that the mTOR mRNA level was decreased in the LV-miR-100-up group compared to the NC group in Jeko-1 cells (*p *= 0.0005). **d** RT-PCR showed that the mTOR mRNA level was decreased in the LV-miR-100-up group compared to the NC group in Mino cells (*p *= 0.0003). **e** Western blot detected the mTOR protein of Jeko-1 cell lines. **f** The expression of mTOR protein in the LV-miR-100-up and LV-mTOR-RNAi groups was decreased compared to the control group and NC group in Jeko-1 cells. **g** Western blot detected the mTOR protein of Mino cell lines. **h** The expression of mTOR protein in the LV-miR-100-up and LV-mTOR-RNAi groups was decreased compared to the control group and NC group in Mino cells

LV-miR-100-up was successfully transfected into Jeko-1 and Mino cells, and miR-100 mRNA levels were remarkably elevated in Jeko-1 and Mino cells (*p *= 0.0003, Fig. 3a, p = 0.0013, Fig. 3b) as determined by RT-PCR. mTOR mRNA levels were lower in Jeko-1 and Mino cells than in NC cells (*p *= 0.0005, Fig. 3c, p = 0.0003, Fig. 3d). The mTOR protein of Jeko-1 and Mino cell lines was detected by Western blot after transfection with LV-miR-100-up and LV-mTOR-RNAi (Fig. 3e, g). The expression of mTOR protein was decreased after transfection with LV-miR-100-up (*p* = 0.004, Fig. 3f, p < 0.0001, Fig. 3h) and LV-mTOR-RNAi (*p* = 0.0001, Fig. 3f, p = 0.0002, Fig. 3h) compared to the corresponding NC in Jeko-1 and Mino cells. mTOR mRNA and protein levels were decreased after LV-miR-100-up transfection.

### Promote miR-100 or reduce mTOR inhibited cell proliferation and arrested the cell cycle in MCL cells

**Fig. 4:**
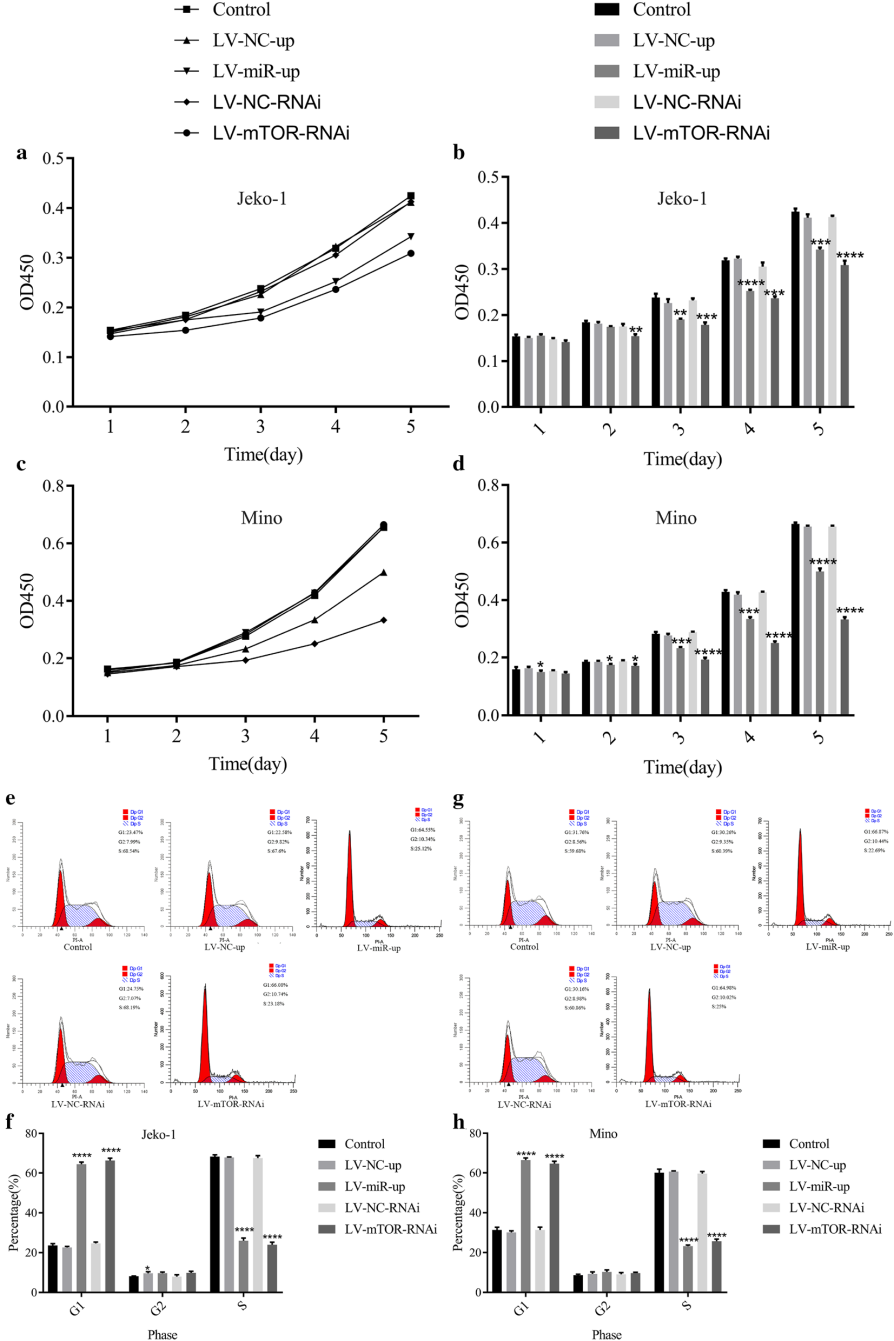
Upregulated miR-100 or downregulated mTOR inhibited cell proliferation and arrested the cell cycle in MCL cells. **a** CCK-8 assay detected the OD value of cell proliferation in Jeko-1 cells. **b** Cell proliferation of Jeko-1 cells in the LV-miR-100-up group (day 3 *p* = 0.0026; day 4 *p* < 0.0001; day 5 *p *= 0.0002) and the LV-mTOR-RNAi group (day 2 *p* = 0.0062; day 3 *p* = 0.0002; day 4 *p *= 0.0003; day 5 *p* < 0.0001) was lower than that in the control and NC groups. **c** CCK-8 assay detected the OD value of the cell proliferation in Mino cells. **d** The data demonstrated that the cell proliferation of Mino cells in the LV-miR-100-up group (day 1 *p* = 0.0424; day 2 *p* = 0.0447; day 3 *p* = 0.0005; day 4 *p* = 0.0001; day 5 *p* < 0.0001) and the LV-mTOR-RNAi group (day 2 *p* = 0.0261; day 3 *p* < 0.0001; day 4 *p* < 0.0001; day 5 *p* < 0.0001) was lower than that in the control and NC groups. **e** Flow cytometry analyzed the cell cycle of Jeko-1 cells. **f** The data showed that the G1 cell cycle rates of Jeko-1 cells in the LV-miR-100-up and LV-mTOR-RNAi groups were higher than those in the NC group and control group, and the Jeko-1 S cell cycle rate in the LV-miR-100-up and LV-mTOR-RNAi groups was lower than those in the NC group and control group (*p* < 0.0001). **g** Flow cytometry analyzed the cell cycle of Mino cells. **h** The data showed that the G1 cell cycle rates of Mino cells in the LV-miR-100-up and LV-mTOR-RNAi groups were higher than those in the NC group and control group, and the S cell cycle rates in the LV-miR-100-up and LV-mTOR-RNAi groups were lower than those in the NC group and control group (*p* < 0.0001)

After transfection with LV-miR-100-up or LV-mTOR-RNAi, the cell proliferation of Jeko-1 and Mino cells was detected by CCK-8 assay, and the cell cycle was checked by flow cytometry. In Jeko-1 cells, the OD values in LV-miR-100-up at day 3 (*p* = 0.0026) and day 5 (*p* = 0.0002) and in LV-mTOR-RNAi at day 2 (*p* = 0.0062) and day 5 (*p* < 0.0001) were lower than those in the control and NC groups (Fig. 4a, b). In Mino cells, the OD value in the LV-miR-100-up group at day 1 (*p* = 0.0424) and day 5 (*p* < 0.0001) and that in LV-mTOR-RNAi at day 2 (*p* = 0.0261) and day 5 (*p* < 0.0001) were lower than that in the control and NC groups (Fig. 4c, d). Flow cytometry analysis showed that the cell cycle rate was checked in Jeko-1 and Mino cells (Fig. 4e, g). This illustrated that the G1 cell cycle rate in the Jeko-1 LV-miR-100-up and LV-mTOR-RNAi groups was higher than that in the NC group. At the same time, the S cell cycle rate in the Jeko-1 LV-miR-100-up and LV-mTOR-RNAi groups was lower than that in the NC group (*p* < 0.0001, Fig. 4f, h). The data demonstrated that upregulation of miR-100 or downregulation of mTOR could inhibit cell proliferation and arrest the cell cycle in MCL cells.

### miR-100-up or mTOR-RNAi induced apoptosis in MCL cells

**Fig. 5:**
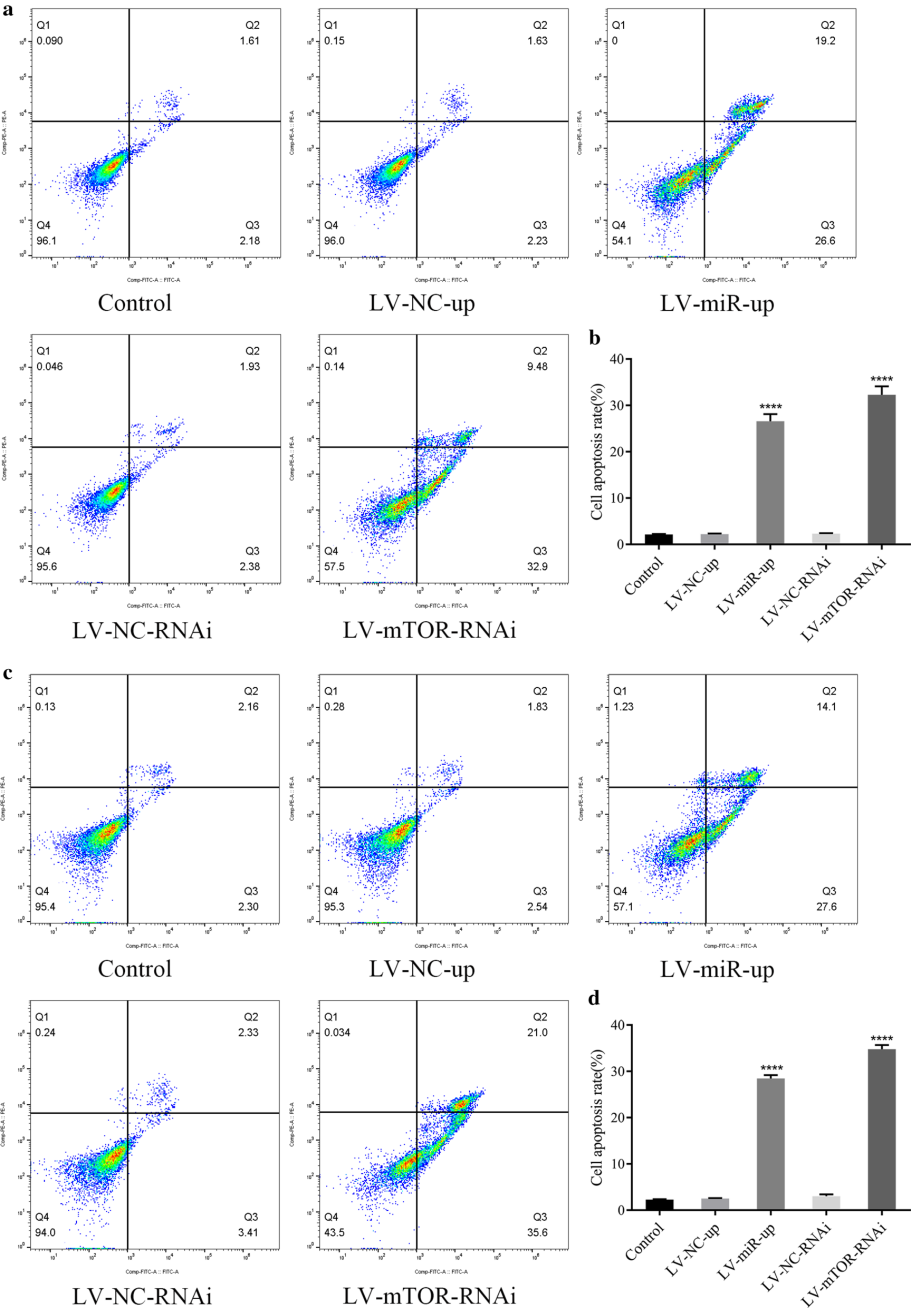
Upregulation of miR-100 and downregulation of mTOR induced apoptosis in MCL cells. **a** Flow cytometry analyzed the apoptosis of Jeko-1 cells. **b** The data showed that the apoptotic rates of the LV-miR-100-up group (*p* < 0.0001) and LV-mTOR-RNAi group (*p* < 0.0001) were higher than those of the NC group and control group in Jeko-1 cells. **c** Flow cytometry analyzed the apoptosis of Mino cells. **d** The data showed that the apoptotic rates of the LV-miR-100-up group (*p* < 0.0001) and LV-mTOR-RNAi group (*p* < 0.0001) were higher than those of the NC group and control group in Mino cells

Apoptotic cells were detected by flow cytometry after transfection with LV-miR-100-up or LV-mTOR-RNAi in Jeko-1 and Mino cells after 6 days (Fig. 5a, c). The apoptotic rates in the LV-miR-100-up (*p* < 0.0001, Fig. 5b, d) and LV-mTOR-RNAi (*p* < 0.0001, Fig. 5b, d) groups were higher than that in the NC group. This result indicated that upregulation of miR‑100 or downregulation of mTOR could induce apoptosis in MCL cells.

### miR-100 targeted mTOR regulation

**Fig. 6:**
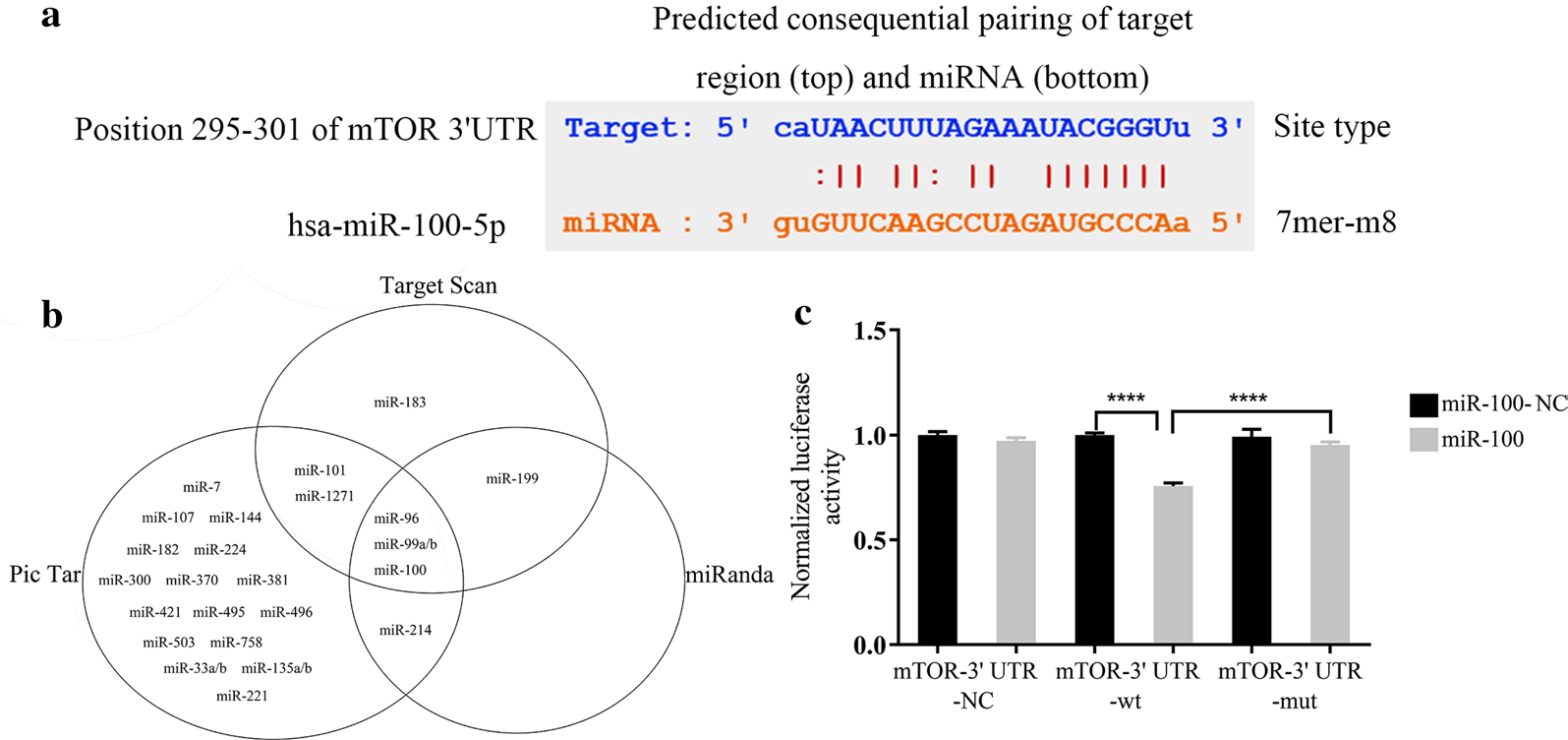
miR‑100 targeted the regulation of mTOR expression. **a** The binding site located between miR-100 and the mTOR-3′ of mTOR mRNA. **b** Prediction of the miRNA targeting the mTOR gene using bioinformatics software. **c** The dual‑luciferase assay showed that the mTOR-3′UTR-wt + miR-100 group significantly decreased the relative luciferase activity of HEK293T cells compared to the mTOR-3′ UTR-mut + miR-100 group (*p* < 0.0001).

To clarify the downstream target of miR-100, we used online Target Scan software, miRanda software, and Pic Tar software to analyze the possible target genes (Fig. 6b). MicroRNA.org online prediction revealed that there was a targeting binding sequence between miR-100 and mTOR (Fig. 6a). Dual luciferase reporter analysis was conducted to detect the binding of miR-100 to the 3′-UTR mTOR mRNA. The WT group was the 3′-UTR wild type, and the MUT group was the 3′-UTR predicted binding site mutation. The study showed that the luciferase activity of the 3′-UTR wild type could be inhibited by miR-100 up (*p* < 0.05), while the luciferase activity of the 3′-UTR mutant remained unaffected (*p* > 0.05, Fig. 6c).

**Fig. 7:**
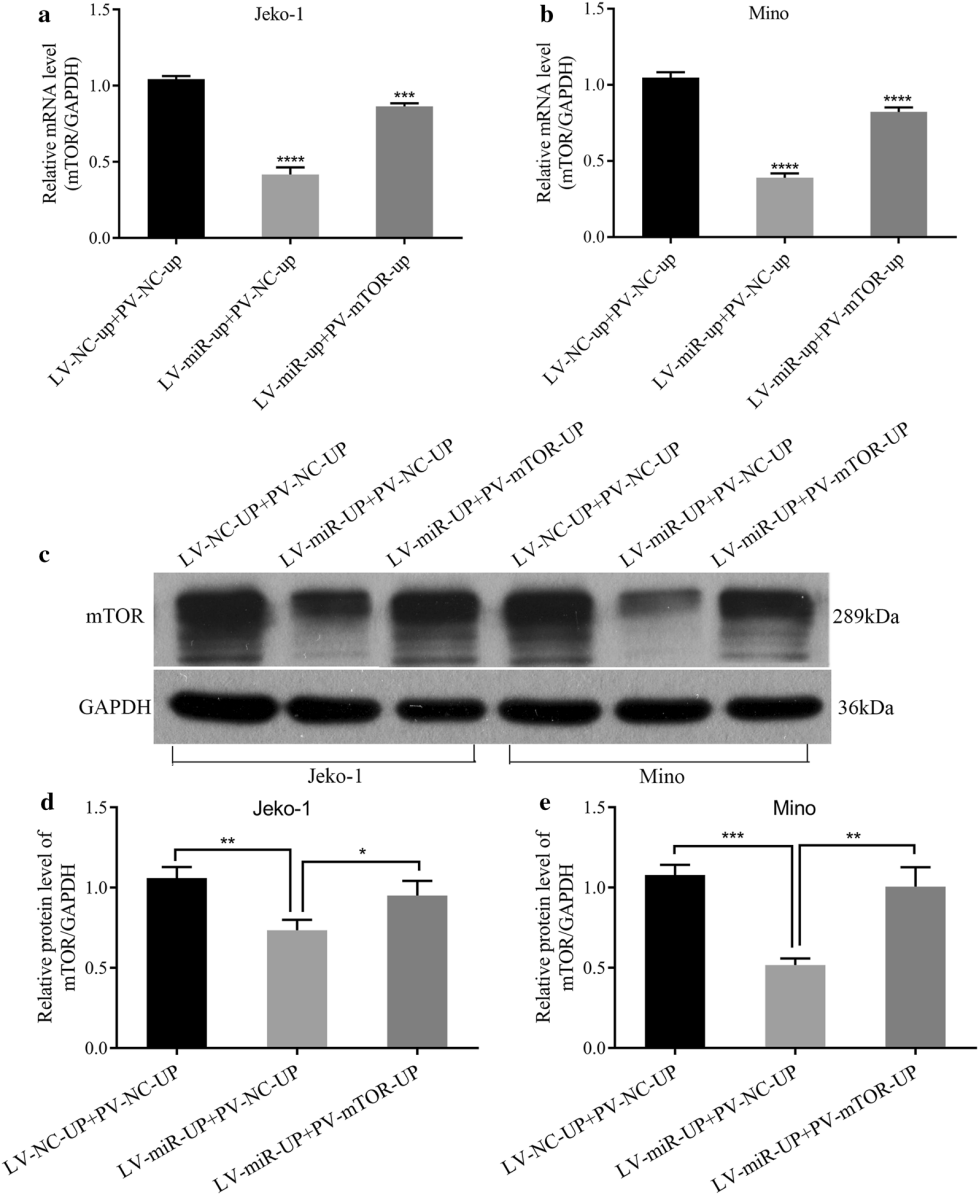
Upregulation of mTOR partially canceled the effects of miR-100 on decreasing the expression of mTOR mRNA and protein. **a** RT-PCR showed that the effects of miR‐100 on decreasing the expression of mTOR mRNA could be partially influenced by overexpression of mTOR in Jeko-1 cells (*p* < 0.0001). **b** RT-PCR showed that the effects of miR‐100 on decreasing the expression of mTOR mRNA could be partially influenced by overexpression of mTOR in Mino cells (*p* < 0.0001). **c** Western blot analysis of mTOR protein after PV-mTOR-up and LV-miR-100-up were cotransferred into the MCL cell lines. **d** The expression of mTOR protein in the LV-miR-up+PV-mTOR-up group was increased compared to the LV-miR-up+PV-NC-up group in Jeko-1 cells (*p* = 0.0287). **e** The expression of mTOR protein in the LV-miR-up+PV-mTOR-up group was increased compared to the LV-miR-up+PV-NC-up group in Mino cells (*p* = 0.0027).

To investigate the effects of the interaction between miR-100 and mTOR in MCL, PV-mTOR-up and LV-miR-100-up were co-transfected into the Jeko-1 and Mino cell lines, respectively. After 6 days, mTOR mRNA was downregulated in the LV-miR-up + PV-NC-up group compared to the control (*p* < 0.0001, Fig. 7a, b), and it was upregulated in the LV-miR-up + PV-mTOR-up group (*p *= 0.0001, Fig. 7a, p < 0.0001, Fig. 7b) compared to the LV-miR-up + PV-NC-up group in Jeko-1 and Mino cells. The expression of mTOR protein is shown in Fig. 7c. The data illustrated that mTOR protein was downregulated in the LV-miR-up + PV-NC-up group compared to the control (p = 0.0041, Fig. 7d, p = 0.0002, Fig. 7e) and was upregulated in the LV-miR-up + PV-mTOR-up group (p = 0.0287, Fig. 7d, p = 0.0027, Fig. 7e) compared to the LV-miR-up + PV-NC-up group. These results showed that the effects of miR-100 on decreasing the expression of mTOR mRNA and protein could be partially affected by the overexpression of mTOR in MCL cells.

### Upregulation of mTOR partially canceled the effects of miR-100 on inhibiting proliferation, inducing apoptosis and arresting the cell cycle in MCL cells

**Fig. 8:**
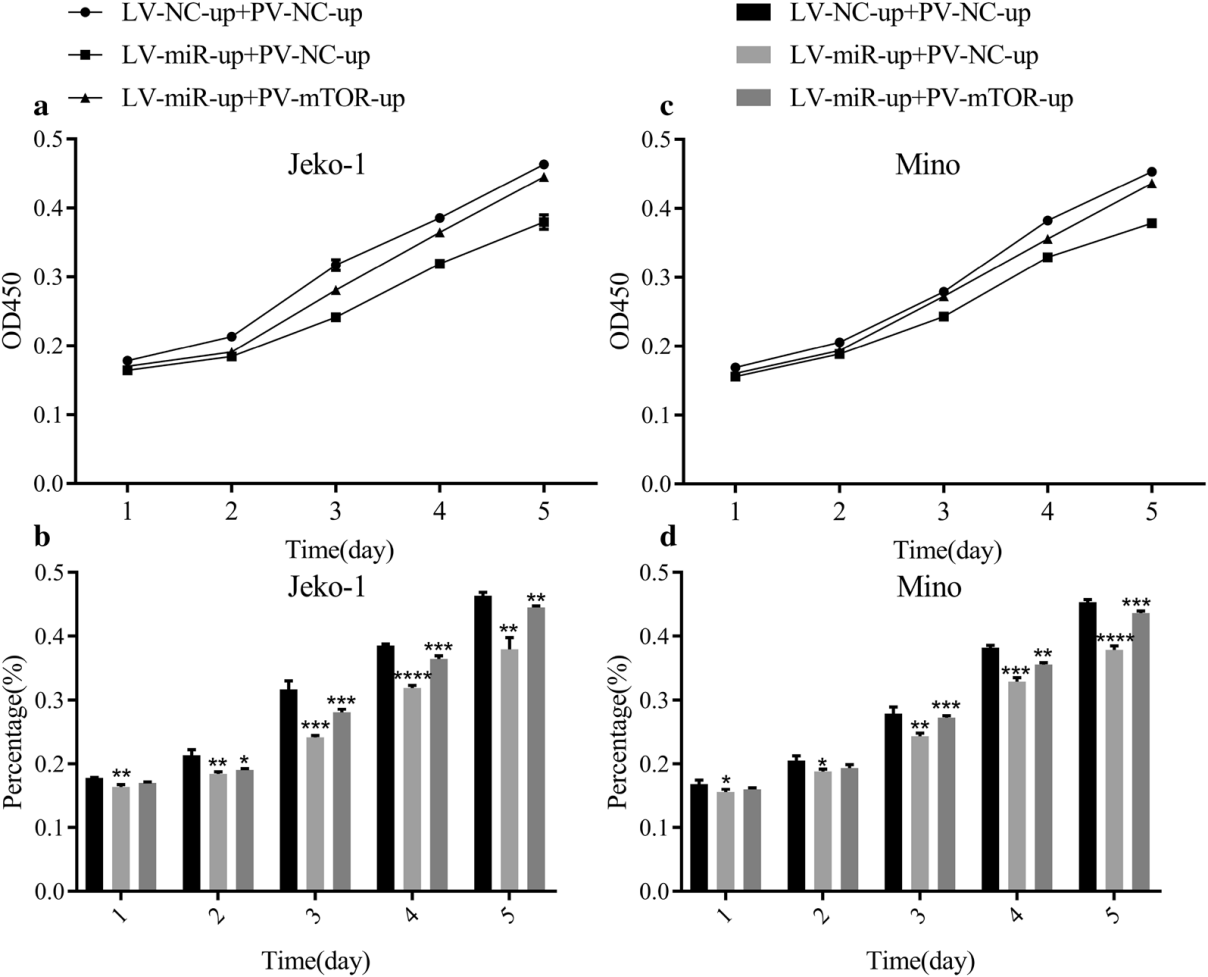
Upregulation of mTOR partially canceled the effects of miR-100 on inhibiting proliferation in MCL cells. **a** CCK-8 assay detected the OD value of Jeko-1 cell proliferation after cotransfection with LV-miR-100-up and PV-mTOR-up. **b** The data demonstrated that the effects of miR‐100 on inhibiting Jeko-1 cell proliferation could be partially influenced by overexpression of mTOR (*p* < 0.05). **c** CCK-8 assay detected the OD value of the Mino cell proliferation after cotransfection with LV-miR-100-up and PV-mTOR-up. **d** The data demonstrated that the effects of miR‐100 on inhibiting Mino cell proliferation could be partially influenced by the overexpression of mTOR (*p* < 0.05)

PV-mTOR-up and LV-miR-100-up were co-transfected into the Jeko-1 and Mino cell lines, respectively. The OD data showed that upregulation of miR-100 and mTOR simultaneously increased cell growth compared with the LV-miR-up + PV-NC-up group in Jeko-1 and Mino cells (Fig. 8a–d).

**Fig. 9:**
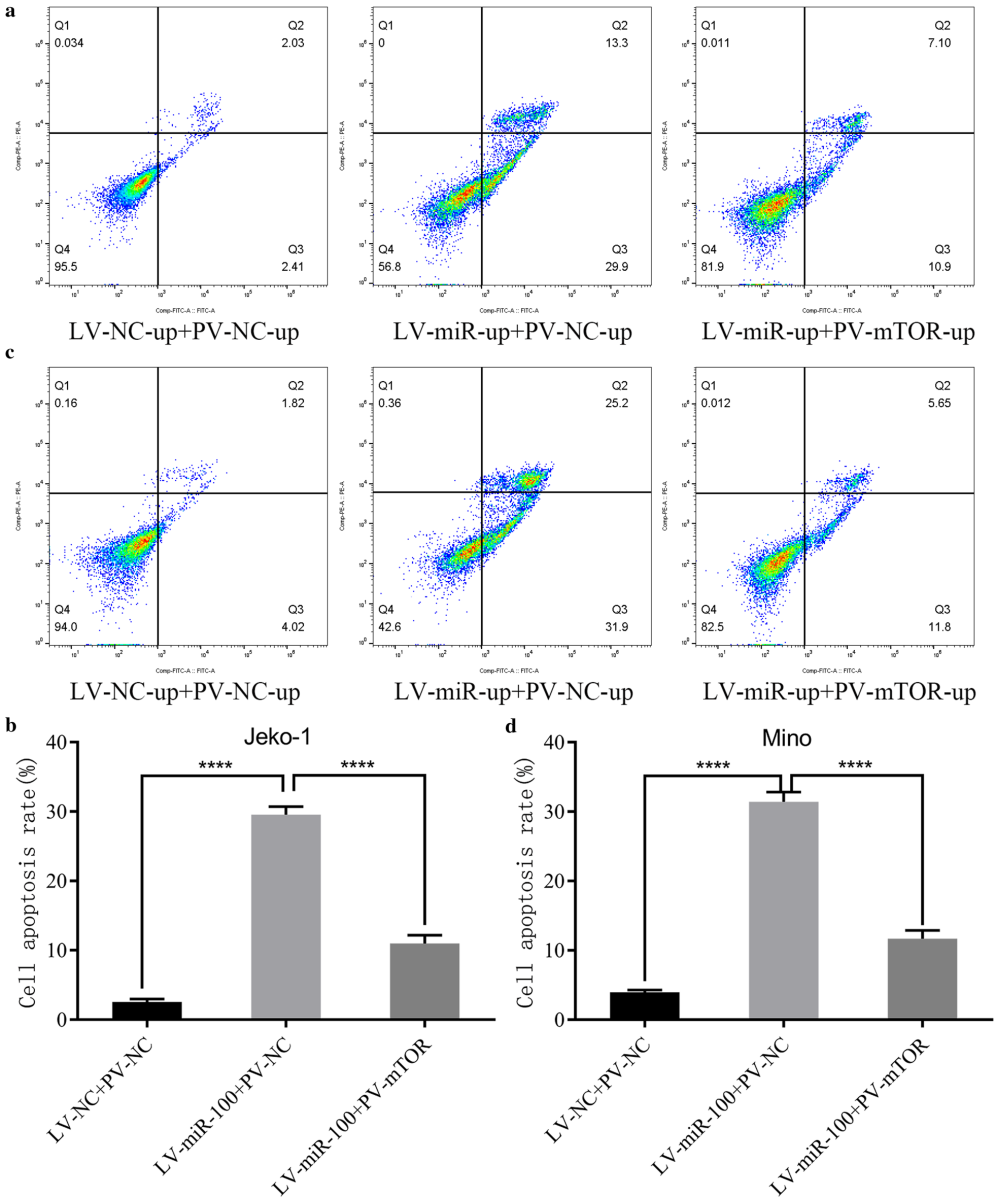
Upregulation of mTOR partially canceled the effects of miR-100 on inducing apoptosis in MCL cells. **a** Flow cytometry analyzed the apoptosis of Jeko-1 cells after cotransfection with LV-miR-100-up and PV-mTOR-up. **b** The data showed that the effects of miR‐100 on inducing apoptosis could be partially influenced by the overexpression of mTOR in Jeko-1 cells (*p* < 0.0001). **c** Flow cytometry analyzed the apoptosis of Mino cells after cotransfection with LV-miR-100-up and PV-mTOR-up. **d** The data showed that the effects of miR‐100 on inducing apoptosis could be partially influenced by the overexpression of mTOR in Mino cells (*p* < 0.0001)

After cotransfection with PV-mTOR-up and LV-miR-100-up into Jeko-1 and Mino cells for 6 days, cell apoptosis was lower than that of the LV-miR-up + PV-NC-up group (Fig. 9a–d).

**Fig. 10:**
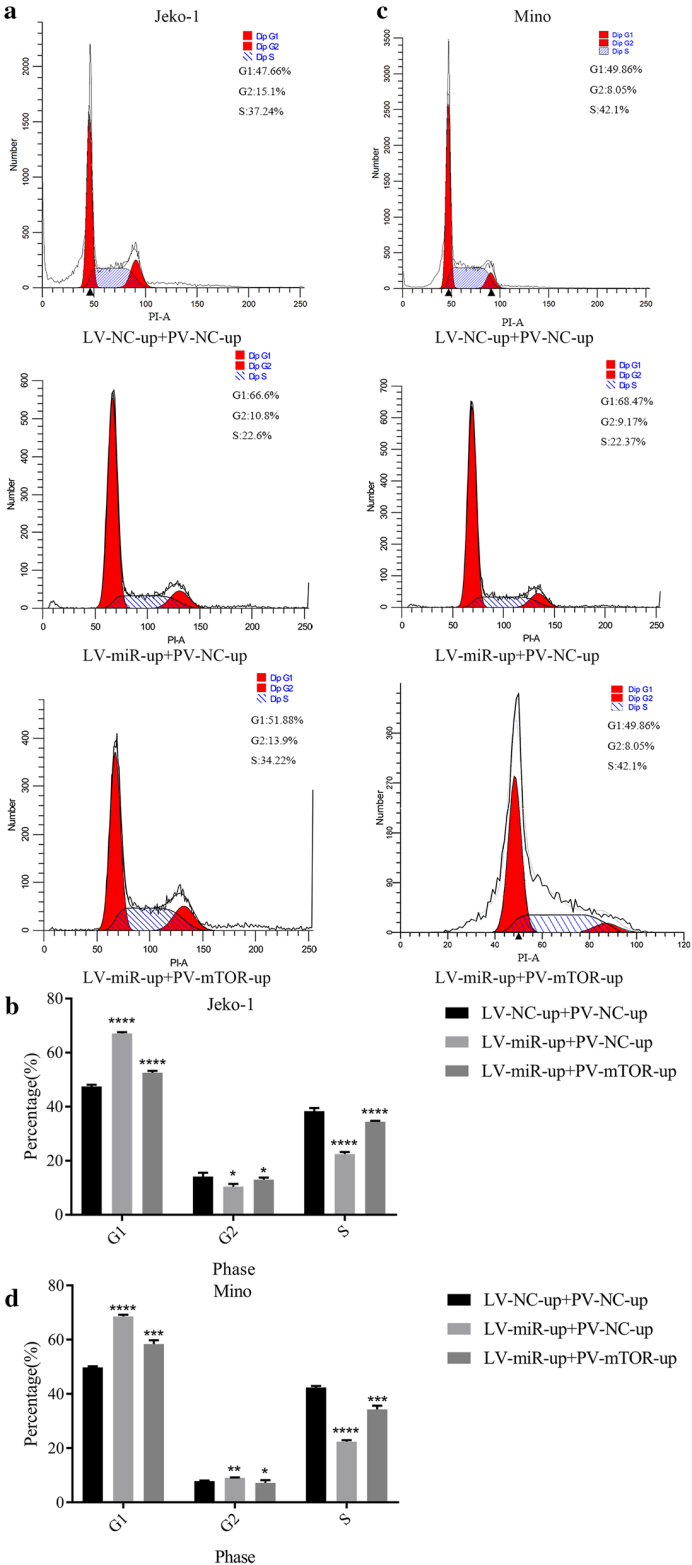
Upregulation of mTOR partially canceled the effects of miR-100 on arresting the cell cycle in MCL cells. **a** Flow cytometry analyzed the cell cycle of Jeko-1 cells after cotransfection with LV-miR-100-up and PV-mTOR-up. **b** The data showed that the effects of miR‐100 on arresting the cell cycle could be partially influenced by the overexpression of mTOR in Jeko-1 cells (*p* < 0.05). **c** Flow cytometry analyzed the cell cycle of Mino cells after cotransfection with LV-miR-100-up and PV-mTOR-up. **d** The data showed that the effects of miR‐100 on arresting the cell cycle could be partially influenced by the overexpression of mTOR in Mino cells (*p* < 0.05)

After cotransfection with LV-miR-100-up and PV-mTOR-up, the G1 cell cycle rate in the Jeko-1 LV-miR-up + PV-mTOR-up group was lower than that in the LV-miR-up + PV-NC-up group, but the S cell cycle rate was higher (Jeko-1: *p* < 0.0001, Fig. 10a, b; Mino: G1 *p* = 0.0003, S *p* = 0.0001, Fig. 10c, d).

## Discussion

Mantle cell lymphoma (MCL) is an aggressive B-cell lymphoma. It is a disease that usually presents at a late stage and is associated with a poor prognosis. While high response rates are observed with induction chemo-immunotherapy, relapse is unfortunately universal in daily clinical practice. Management of the disease with a lack of efficiency is still challenging. Drug resistance is a multifactorial process that is responsible for the absence of chemo-response in primary and secondary types of tumors. Therefore, it is critical to identify novel targets for MCL therapy.

miRNAs are a class of small, endogenous, single-strand noncoding RNAs with a length of 22 ~ 24 nucleotides that exert their function by binding to the 3′ untranslated region (3′ UTR) of their target mRNAs [10]. miRNAs regulate the expression of > 30% of human target genes by degradation or inhibition of target gene mRNA translation. Each miRNA is predicted to target hundreds of genes, and each target transcript may interact with multiple miRNAs. Accumulating evidence has revealed that altered expression of miRNAs plays an important role in the tumorigenesis of many human cancers [11–13]. In the meantime, studies have demonstrated that a single cancer can be driven by different miRNAs, while a single miRNA can be aberrantly expressed in different cancers [14–16].

Different kinds of miRNAs have been reported to participate in the physiological processes in MCL by regulating several target genes [17–22]. miR-223 influenced MCL cell proliferation and apoptosis by targeting SOX11 directly [23]. It has been confirmed that abnormal expression of miRNAs affects the proliferation, apoptosis and cell cycle in MCL cells by targeting multiple intracellular signaling pathways, such as the AKT signaling pathway and NF-kB signaling pathway [24, 25]. miR17-92 clusters play a role in the occurrence and development of MCL, such as activating the PI3K/AKT pathway to mediate nesting [26]. In recurrent mantle cell lymphoma (MCL SP) cells and cell lines, the low expression of miR-16 and the high expression of BMI1 could promote tumorigenesis and development by inhibiting apoptosis [27]. As the first tumor-specific inhibitor of miRNAs, MRX34 has entered phase 1 clinical trials in patients with hepatocellular carcinoma and other solid tumors to correct the normal expression of miRNAs or overexpression in tumor cells [28].

miR-100 is a member of the miRNA family. The function of miR-100 is controversial. miR-100 was found to act as a tumor suppressor by deregulating its target genes in hepatocellular carcinoma [29], epithelial ovarian cancer [30], nasopharyngeal carcinoma [31], gastric cancer [32], and breast cancer [33]. However, miR-100 was also found to act as an oncogene in renal cell carcinoma [34] and acute myeloid leukemia [35]. Zhang et al. [36] demonstrated that miR-100 is downregulated in acute lymphoblastic leukemia patients and has different roles in myeloid cell and lymphocyte pathogenesis.

However, no clear information about the function or molecular mechanism of miR-100 in MCL has been reported. In this study, for the first time, we detected the expression of miR-100 in MCL tissues and cell lines, and hyperplastic lymphadenitis and peripheral lymphocyte cells were used as the controls. We demonstrated that the miR-100 level was lower in 18 patients with MCL and in 3 MCL cell lines than the control. Based on the above findings, we further elucidated the molecular mechanism of miR-100 in regulating cell proliferation, apoptosis and cell cycle progression in MCL cell lines by transfecting LV-miR-100-up into Jeko-1 and Mino cells. The overexpression of miR-100 inhibited cell proliferation, induced cell apoptosis and induced G1 arrest in Jeko-1 and Mino cell lines. This suggests that miR-100 might be a tumor suppressor gene in MCL.

At the same time, we detected the expression of mTOR in MCL tissues and cell lines. We demonstrated that the mTOR level was higher in 18 patients with MCL and in 3 MCL cell lines compared to the control. We also found that the expression of mTOR was negatively correlated with miR-100 in MCL tissues and cells. These differences were statistically significant. These results suggest that miR-100 is a tumor suppressor gene and that mTOR might be a candidate oncogene in MCL.

The PI3K-Akt-mTOR pathway is the main regulatory pathway of protein translation synthesis, and mTOR is an important part of this signaling pathway [37]. The overexpression of the PI3K-Akt-mTOR pathway could lead to increased transcription of the genes encoding the proliferation control proteins in cells, resulting in a large number of translation and synthesis of cell proliferation-related proteins, thus promoting cell proliferation and inhibition of cell apoptosis. The molecular biology characteristics of MCL are chromosomal translocation and abnormal cytogenetic t (11; 14) (q13; 32), which leads to upregulation of cyclin D1. Cyclin D1 is a G1 phase cyclin, which drives cells from G1 phase to S phase and promotes cell proliferation, which plays an important role in the occurrence and development in MCL. Gera et al. [38] found that inhibiting mTOR led to downregulation of cyclin D1 transcription and translation and induced G1 arrest. Therefore, we hypothesized that inhibition of the mTOR gene may inhibit the proliferation of tumor cells, induce apoptosis and induce G1 arrest in MCL. Based on the above findings, we transfected LV-mTOR-RNAi into the Jeko-1 and Mino cells. It showed that downregulated mTOR could inhibit cell proliferation, induce cell apoptosis and induce G1 arrest in Jeko-1 and Mino cell lines, which was the same as the upregulation of miR-100 expression. We detected that upregulation miR-100 could increase the expression of mTOR mRNA and protein, which indicated that miR-100 might cooperate with mTOR and play an important role in MCL. These results suggested that MCL cell growth could be influenced by interaction between miR-100 and mTOR.

Combined with these results, we hypothesized that in the development of MCL, the expression of miR-100 decreased, which led to the decrease in mTOR degradation. The increase in mTOR expression promoted MCL cell proliferation, inhibited cell apoptosis, and increased the number of cells in G1 phase into S phase. Therefore, we further verify whether miR-100 could target mTOR gene in MCL, thus affecting the proliferation, apoptosis and cell cycle of MCL. Using bioinformatical tools, we found that the 3′-UTR of mTOR has a putative binding site for miR-100. Luciferase assays showed that miR-100 inhibited the luciferase activity of wild-type 3′-UTR of mTOR, but not mutant 3′-UTR of mTOR, which confirmed mTOR was a direct target of miR-100. To confirm the above results, a recovery experiment was conducted in MCL cells. Overexpression of mTOR partially canceled the effects of miR-100 upregulation on inhibiting proliferation, promoting apoptosis and arresting the cell cycle in the Jeko-1 and Mino cells.

Therefore, we can infer that the expression of miR-100 in MCL is decreased, and the degradation of mTOR is reduced, which leads to the excessive activation of mTOR, promotes the proliferation of MCL cells, inhibits apoptosis and promotes the transition from G1 phase to S phase. Upregulation of miR-100 can inhibit cell proliferation, induce apoptosis and arrest the cell cycle in the G1 phase by targeting downregulation of mTOR expression. In conclusion, miR-100 plays an important role in the development of MCL by regulating the expression of mTOR, which may become a new target in the treatment of MCL.

## Data Availability

The data-sets used and/or analyzed during the current study are available from the corresponding author on reasonable request.
